# Spatial modelling improves genetic evaluation in smallholder breeding programs

**DOI:** 10.1186/s12711-020-00588-w

**Published:** 2020-11-16

**Authors:** Maria L. Selle, Ingelin Steinsland, Owen Powell, John M. Hickey, Gregor Gorjanc

**Affiliations:** 1 Department of Mathematical Sciences, Norwegian University of Science and Technology, Trondheim, Norway; 2 The Roslin Institute and Royal (Dick) School of Veterinary Studies, University of Edinburgh, Edinburgh, UK

## Abstract

**Background:**

Breeders and geneticists use statistical models to separate genetic and environmental effects on phenotype. A common way to separate these effects is to model a descriptor of an environment, a contemporary group or herd, and account for genetic relationship between animals across environments. However, separating the genetic and environmental effects in smallholder systems is challenging due to small herd sizes and weak genetic connectedness across herds. We hypothesised that accounting for spatial relationships between nearby herds can improve genetic evaluation in smallholder systems. Furthermore, geographically referenced environmental covariates are increasingly available and could model underlying sources of spatial relationships. The objective of this study was therefore, to evaluate the potential of spatial modelling to improve genetic evaluation in dairy cattle smallholder systems.

**Methods:**

We performed simulations and real dairy cattle data analysis to test our hypothesis. We modelled environmental variation by estimating herd and spatial effects. Herd effects were considered independent, whereas spatial effects had distance-based covariance between herds. We compared these models using pedigree or genomic data.

**Results:**

The results show that in smallholder systems (i) standard models do not separate genetic and environmental effects accurately, (ii) spatial modelling increases the accuracy of genetic evaluation for phenotyped and non-phenotyped animals, (iii) environmental covariates do not substantially improve the accuracy of genetic evaluation beyond simple distance-based relationships between herds, (iv) the benefit of spatial modelling was largest when separating the genetic and environmental effects was challenging, and (v) spatial modelling was beneficial when using either pedigree or genomic data.

**Conclusions:**

We have demonstrated the potential of spatial modelling to improve genetic evaluation in smallholder systems. This improvement is driven by establishing environmental connectedness between herds, which enhances separation of genetic and environmental effects. We suggest routine spatial modelling in genetic evaluations, particularly for smallholder systems. Spatial modelling could also have a major impact in studies of human and wild populations.

## Background

This study evaluates the potential of spatial modelling to improve the genetic evaluation of animals in smallholder systems. Over the past century, genetic selection of dairy cattle has significantly increased milk production in developed countries [1]. For example, the average milk production of US Holstein cows has almost doubled between 1960 and 2000, and more than half of this is due to genetic improvement [2]. However, such improvements have not been achieved in low to middle income countries, for example, in East Africa. For instance, Rademaker et al [3] reported that milk yield in smallholder farms in Kenya are about 5 to 8 L per cow per day, which is several-fold smaller than in large-scale commercial farmers around the world. These low milk yields are due to environmental, technological and infrastructural difficulties as well as mixed breed composition [4, 5]. Whereas large-scale commercial farmers measure phenotypes accurately, and keep records of performance and pedigree, smallholders usually do not keep records and the absence of routine phenotyping systems reduces the accuracy of these records [6, 7].

To perform an accurate genetic evaluation of animals in a breeding program, a sufficient amount of data is needed, and the data should be appropriately structured [7–9]. In developed countries, a small number of large-scale commercial farms produce most of the milk, and there is a widespread use of artificial insemination that establishes strong genetic connectedness between herds. However, in many smallholder systems, smallholder farms contribute significantly to milk production, and there is low usage of artificial insemination with consequent weak genetic connectedness between herds. For example, smallholder milk-producing households in Kenya with one to three cows represent the majority of the national dairy population [3, 10]. Furthermore, 87% of surveyed Kenyan farmers used natural mating services rather than artificial insemination, even though 54% reported that they would have preferred artificial insemination [11]. Similar proportions were reported elsewhere [12, 13].

Small herd sizes and weak genetic connectedness between herds challenge accurate genetic evaluation [14–16]. When herds are small, it is difficult to accurately separate the genetic and environmental effects on the phenotype. Furthermore, with weak genetic connectedness, low relationships between animals in different herds limit sharing of information, which additionally limits accurate separation of the genetic and environmental effects. Since most smallholders mate cows with their own or neighbour’s bull, it is reasonable to assume that most farmers in close distance use the same bulls. This system genetically connects herds that are close in distance although the overall genetic connectedness across the country is weak.

In the statistical models for genetic evaluations, the genetic effect is modelled using expected or realised genetic relationship between animals, respectively derived from pedigree or genomic data. A herd effect, or a herd-year-season effect, is often included as the main environmental effect [6, 17–20]. When herd sizes are small, the herd effects are treated as random to increase sharing of information between herds and increase accuracy compared to treating them as fixed [7, 18, 21, 22]. In the extreme case of a single animal per herd, modelling herds as random is, in fact, the only possible approach [7]. In addition, including other factors and covariates in the statistical models is a way of including information in the model that can further enhance the separation of genetic and environmental effects.

Environmental effects can be on management (herd) level, or a larger scale, likely shared by herds in close distance. Examples of environmental effects on management level are education, age, and experience of the farmer, use of natural mating or artificial insemination etc. Some of these effects can be similar for herds in proximity. Feed quality is likely similar in nearby farms and veterinary practices are likely to vary with local, regional or national government policies. Farmers with higher levels of education and experience will likely be more skilled and positively affect phenotype. Age is usually also related to experience. Examples of large-scale environmental effects are climate effects, proximity to roads, markets and towns, and government policies. Many of the environmental effects can be assumed to be spatially correlated. We will refer to the environmental effects on management level as herd effects, and the large-scale environmental effects as spatial effects.

There are multiple spatial models that could be used in an animal breeding context. A prerequisite for this is that data are geographically referenced. Geographical location can be described coarsely with regions or precisely with point coordinates. For an application of region-based models in an animal breeding context see [23], where veterinary district was modelled  as an environmental effect with covariance between neighbouring districts [24, 25]. We focus on coordinate-based models (often referred to as geostatistical models [25–28]) to account for fine-grained spatial relationships between smallholder farms. The only requirement for a coordinate-based model is that we collect herd coordinates and then all data pertaining to a herd is point-referenced. For a herd *i*, we define a tuple ${\mathbf {w}}_i$ that typically contains two-dimensional coordinates (latitude and longitude), but note that further extensions are possible [29, 30]. The observation at specific locations and locations themselves can vary continuously over a geographical region. A common model for such continuous spatial processes is a Gaussian random field where we model observations at a set of locations $(y({\mathbf {w}}_1),...,y({\mathbf {w}}_n))$ with a multivariate normal distribution with mean ${\mu }$ and a distance based covariance matrix ${\Sigma }$ [25]. The same approach can also be used as a model component in the context of a linear mixed model [25], as is the case with genetic effects, but in the spatial context, we account for relationships between locations. There are multiple possible covariance functions for spatial modelling. Most of them assume stationarity and isotropy, so that ${\mu }({\mathbf {w}}) = {\mu }$ and spatial covariance between locations is a function of Euclidian distance between locations and model parameters, such as variance. The most commonly used is the Matérn covariance function [31].

Modelling with continuously indexed Gaussian random fields is computationally challenging because they give rise to dense precision (covariance inverse) matrices that are numerically expensive to factorise [25], as is the case with genomic models [32, 33]. Gaussian Markov random fields approximate Gaussian random fields by assuming conditional independence, which increases sparsity of the precision matrix and reduces computational complexity. Lindgren et al. [29] showed how to construct an explicit link between some Gaussian random fields and Gaussian Markov random fields via a solution of stochastic partial differential equations. They also proposed use of a finite element method to further reduce computational complexity. This approach allows the implementation of computationally efficient numerical methods for spatial modelling of large-scale point-referenced data. Assuming conditional independence to scale genomic modelling has also been proposed recently [34, 35].

This study aimed at evaluating the potential of spatial modelling in addition to modelling independent herd effects to improve genetic evaluation in smallholder systems, and to determine if the impact depended on the genetic connectedness across the herds, and the use of pedigree or genomic data. In addition, we tested whether adding environmental covariates was beneficial beyond the simple distance-based relationships between herds.

We performed a simulation study that resembled smallholder systems that are commonly observed in East Africa with small herd sizes. We evaluated scenarios with different genetic connectedness across herds, herd distribution and spatial variation. The results showed that spatial modelling improved genetic evaluations, especially with weak genetic connectedness. We also analysed real dairy cattle data and the results indicated that the standard and spatial models separated the genetic and environmental effects in different ways for animals living in areas with larger spatial effects.

## Material and methods

We first introduce the data used in the analyses; a simulated smallholder dairy cattle data, and a real dairy cattle data. Then, we present the statistical models used for genetic evaluation and how we fitted and evaluated the models. Scripts for data simulation and model fitting are available in Additional file 1.

### Simulation

**Fig. 1:**
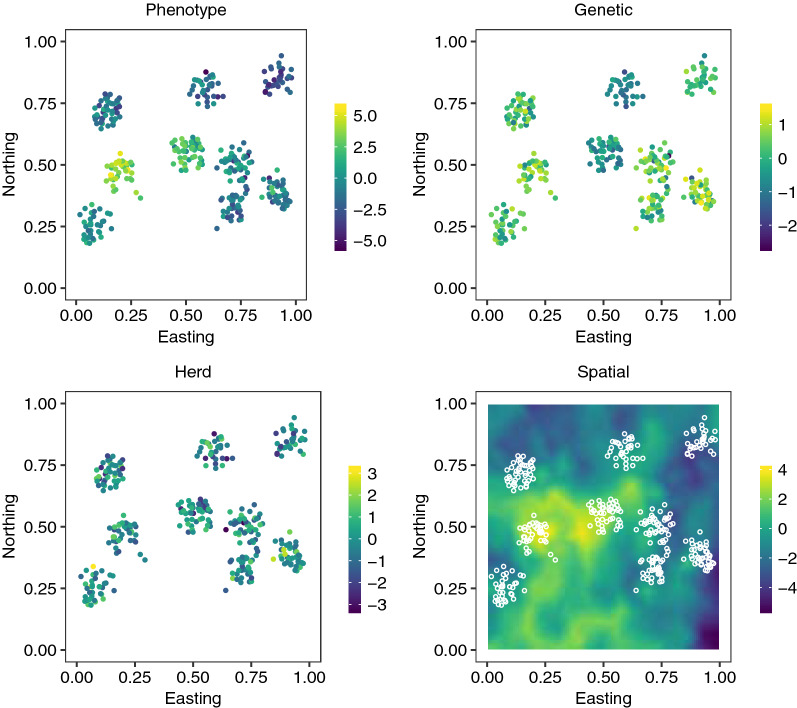
Illustration of the simulation. Each point denotes an animal, their location in a country and colour of the point denotes value of phenotype and underlying genetic, herd and spatial effects (residual not shown)

We used simulation to evaluate the potential of spatial modelling to improve genetic evaluation. The simulated data resembled the smallholder systems commonly observed in East Africa with small herds clustered in villages and a varying level of genetic connectedness. We simulated phenotype observations $y_i$ as:

1 $$\begin{aligned} y_i = \mu + g_i + h_i + s_i + e_i, \end{aligned}$$

where $\mu$ is population mean, $g_i$ is the additive genetic effect of individual *i*, $h_i \sim {\mathcal {N}}(0, \sigma _{h}^2)$ is the herd effect with $\sigma _{h}^2 =0.25$, $s_i$ is the spatial effect, and $e_i \sim {\mathcal {N}}(0,\sigma _{e}^2)$ is an independent residual with $\sigma _{e}^2 = 0.25$. Below, we describe the simulation of genetic and spatial effects. In Fig. 1, we show a conceptual illustration of the simulation. The top left panel shows the phenotypes, and the remaining panels show the genetic, herd and spatial effects. Note the most bottom-right village (cluster) with high genetic merit animals, but intermediate phenotypes due to negative spatial effects.

We simulated the data under three scenarios of genetic connectedness, from weak genetic connectedness between herds from different villages to strong genetic connectedness across all herds regardless of the village. We generated 60 independent data sets for each scenario of genetic connectedness.

#### Simulation of founders

First, we simulated a genome consisting of 10 chromosome pairs with cattle genome and demography parameters [36]. To this end, we used the Markovian Coalescent Simulator [37] and AlphaSimR [38, 39] to simulate genome sequences for 5000 founder individuals, which served as the initial parents. For each chromosome, we randomly chose segregating sites in the founders’ sequences to serve as 5000 single-nucleotide polymorphisms (SNPs) and 1000 quantitative trait loci (QTL) per chromosome, yielding 50,000 SNPs and 10,000 QTL.

Then, we simulated a single complex trait with additive architecture by sampling QTL allele substitution effects from a standard normal distribution. We multiplied these with individuals’ QTL and summed them to the true breeding value. Then we simulated phenotypes with different heritabilities for cows ($h^2=0.3$) and bulls ($h^2=0.8$) to reflect different amounts of information per gender. These phenotypes were used for the initial assignment of bulls and their selection throughout the evaluation phase.

#### Population simulation

We created 100 villages, each consisting of 20 herds, with herd sizes generated from a zero truncated Poisson distribution with parameter $\lambda =1.5$. The 110 best males from the founder individuals (based on true genetic values) were assigned as breeding bulls, 100 as natural mating or artificial insemination bulls depending on the scenario, and 10 as artificial insemination bulls. The remaining founders were considered as cows and were randomly placed in the herds. Since the herd sizes were sampled, we did not have the same number of individuals in each replicate. On average, there were 3860 cows in total, and the cows not assigned to a herd were discarded.

We positioned the 100 villages by assuming a square country and sampled village coordinates in the north-south and east-west direction from a uniform distribution on (0, 1). We then positioned the 2000 herds by sampling their coordinates ${\mathbf {w}}\in {\mathbb {R}}^2$ from a bi-variate normal distribution with mean from the corresponding village coordinates and location variance $3.5\cdot 10^{-4} {\mathbf {I}}_{2\times 2}$. This clustered the herds around village centres. We chose the location variance to achieve reasonable spread and clustering. We tested the sensitivity of results to this simulation parameter.

We tested three levels of genetic connectedness by controlling the breeding strategy. To achieve weak genetic connectedness, each village used their own natural mating bull, meaning that the cows were strongly related within the village and nominally unrelated between villages. However, there was still some base level genetic relationship due to the shared population history. To achieve intermediate genetic connectedness, each village used their own bull for mating in 75% of the herds, while the remaining herds in the village used one of the ten artificial insemination bulls at random, meaning that cows were still strongly related within villages, and somewhat related between villages. To achieve strong genetic connectedness, 100 artificial insemination bulls were randomly mated to cows across all herds and villages, meaning that cows were equally related within and between villages. For this last scenario, we used the 100 artificial insemination bulls instead of the ten artificial insemination bulls in order to maintain a relatively high degree of genetic diversity, and with this, a more challenging situation for separation of environmental and genetic effects.

The three scenarios were then simulated over 12 discrete generations. Within each farm, we replaced the current cows by their newborn female calves. The cows with male calves were not replaced, and their calves were candidates for natural mating if they came from a farm using natural mating, or for artificial insemination if they came from a farm using artificial insemination.

In the 11th generation, we scaled the true breeding values to have mean 0 and variance $\sigma _g^2=0.1$, and used them as genetic effects in the model for phenotype observation $y_i$ in Eq. (1), with 3860 records on average. In addition, the female calves in the 12th generation were kept for prediction purposes. To ease the computations with the genome-based model, we predicted breeding values for randomly chosen 200 calves in the 12th generation.

#### Simulation of spatial effects

We simulated spatial effects from multiple Gaussian random fields to mimic several sources of environmental effects. We imagined that these different sources could be temperature, precipitation, elevation, land size, proximity to markets and towns, availability of extension services, vaccine use, local and regional policies etc. We simulated the effects of eight such processes ${\mathbf {v}}_k, k = 1,\ldots ,8$ at the herd locations from a Gaussian random field with mean 0 and a Matérn covariance function [31]. The Matérn covariance function between locations ${\mathbf {w}}_i, {\mathbf {w}}_j \in {\mathbb {R}}^d$ is:

2 $$\begin{aligned} Cov({\mathbf {w}}_i, {\mathbf {w}}_j) = \frac{\sigma ^2}{2^{\nu -1} \Gamma (\nu )}\left( \kappa \Vert {\mathbf {w}}_j - {\mathbf {w}}_i \Vert \right) ^{\nu } K_{\nu }\left( \kappa \Vert {\mathbf {w}}_j - {\mathbf {w}}_i \Vert \right) , \end{aligned}$$

where $K_{\nu }$ is the modified Bessel function of the second kind and the order $\nu >0$ determines the mean-square differentiability of the field. The parameter $\kappa$ can be expressed as $\kappa = \sqrt{8\nu }/\rho$, where $\rho >0$ is the range parameter describing the distance where correlation between two points is near 0.1, and $\sigma ^2$ is the marginal variance. We varied these parameters to simulate processes on large and small scales and with different properties. Specifically, we sampled the range parameter $\rho$ for each of the processes ${\mathbf {v}}_k$ from a uniform distribution on (0.1, 0.5), set the marginal variance $\sigma ^2$ to either 0.2 or 0.3 with equal probability, and fixed the parameter $\nu$ to 1.

We finally summed the eight  processes to obtain the total spatial effect (Fig. 1) for all herd locations ${\mathbf {s}}$, with ${\mathbf {s}}({\mathbf {w}}_i)$ being the total spatial effect at location ${\mathbf {w}}_i$. We differentially emphasised some processes according to:

$$\begin{aligned} {\mathbf {s}} = \sum _{k=1}^3 {\mathbf {v}}_k + \sum _{k=4}^6 {\mathbf {v}}_k (1 + \alpha _k) + \sum _{k=7}^8 {\mathbf {v}}_k (1 + \alpha _k + \beta _k ) \end{aligned}$$

with the weights $\alpha ,\beta \sim$Uniform$(-\,0.5,0.5)$. We scaled the spatial effects to have mean 0 and variance $\sigma _{s}^2=0.4$.

#### Environmental covariates

We assumed that some spatial processes could be observed as environmental covariates at herd locations, possibly with some noise. We took the eight real processes and sampled two more (with mean 0 and a Matérn covariance function) that did not affect the phenotype.

For the spatial processes ${\mathbf {v}}_1$, ${\mathbf {v}}_2$, and ${\mathbf {v}}_3$, we assumed that we could observe the spatial covariates perfectly without error, which could be reasonable for some covariates, such as temperature and precipitation.

For the spatial processes ${\mathbf {v}}_4$, ${\mathbf {v}}_5$, and ${\mathbf {v}}_6$, we assumed that we could  not observe them accurately, so we added normal distributed error with mean 0 and variance equal to 10% of the process marginal variance. This could be reasonable for some covariates that are difficult to measure or that vary with time; it could, for example, be challenging to quantify the amount and quality of feed.

For the spatial processes ${\mathbf {v}}_7$ and ${\mathbf {v}}_8$, we assumed that we could only observe categorical realisations of the continuous effects, for example, distance to markets and towns could be categorised as either a rural or urban area. For the process ${\mathbf{v}}_7$, we created a two-level factor by sampling a threshold from a uniform distribution between one standard deviation from the mean of ${\mathbf {v}}_7$ in both negative and positive directions. Values of ${\mathbf {v}}_7$ above the threshold were assigned one level, and values below were assigned the other level. For the process ${\mathbf {v}}_8$, we created a three-level factor by sampling two thresholds. The lower threshold was sampled from a uniform distribution between two standard deviations below the mean of ${\mathbf {v}}_8$ and the mean of ${\mathbf {v}}_8$. The upper threshold was sampled from a uniform distribution between the mean of ${\mathbf {v}}_8$ and two standard deviations above the mean of ${\mathbf {v}}_8$. The values of ${\mathbf {v}}_8$ were then assigned one of three levels depending on thresholds.

#### Changing the proportion of spatial variance and herd clustering

To evaluate how the models performed when there was no or little spatial effect on the phenotype, we created scenarios with different proportions of spatial variance relative to the sum of herd effect variance and spatial variance so that the total variation between herds was constant. We kept $\sigma _{s}^2 + \sigma _{h}^2 = 0.65$, and let $\sigma _{s}^2/(\sigma _{s}^2+\sigma _h^2) = \{0, 0.2,0.4,0.6,0.8,1\}$. This was repeated for 30 of the data sets.

We also evaluated the importance of how tightly the herds were clustered around village centres. We varied the location variance of the bi-variate distribution for the herd coordinates ${\mathbf {w}}\in {\mathbb {R}}^2$ from $1.0\cdot 10^{-4} {\mathbf {I}}_{2\times 2}$ (strong clustering), $3.5\cdot 10^{-4} {\mathbf {I}}_{2\times 2}$ (intermediate clustering) to $9.0\cdot 10^{-4} {\mathbf {I}}_{2\times 2}$ (weak clustering). This was repeated for each of the 60 data sets.

### Real dairy cattle data

We then analysed phenotypic data for 30,314 Brown-Swiss cattle data from Slovenia collected between 2004 and 2019, from 2012 herds. The data included a body conformation measure, year and scorer, cow’s age, stage of lactation, year and month of calving, herd and the farm’s coordinates. In addition, the data contained a pedigree for 56,465 animals including the phenotyped cows. We analysed the body conformation, which we standardised by subtracting the phenotypic mean and dividing by the phenotypic standard deviation.

The average herd size was approximately 15 cows per herd, and most cows were in herds with more than five animals. To imitate data typical of smallholder systems, with few individuals per herd, we used a subset of the full data. We sampled 3800 individuals without replacement, with sampling probability equal to the inverse herd size, meaning that larger herds had fewer records in the data subset. The subset contained cows from 1838 herds, and the average herd size was about 2 cows per herd. The herds were spread over most of Slovenia (see Additional file 2: Figure S2).

### Statistical models

The following model was fitted to the observed phenotype $y_i$ of individual $i=1,...,n$:

3 $$\begin{aligned} y_i = {\mathbf {x}}_i {\beta } + a_i + h_i + s_i + e_i, \end{aligned}$$

where ${\beta }$ is a vector containing contemporary group effects, including a common intercept, with known covariate vector ${\mathbf {x}}_i$ and $\beta \sim {\mathcal {N}}(0,\sigma ^2_{\beta })$, $a_i$ is the additive genetic effect (breeding value), $h_i$ is the herd effect with ${\mathbf {h}} \sim {\mathcal {N}}({\mathbf {0}}, {\mathbf {I}}\sigma ^2_h)$, $s_i$ is the spatial effect for the herd at location ${\mathbf {w}}_i \in {\mathbb {R}}^2$ modelled with a Gaussian Markov random field with ${\mu }={\mathbf {0}}$ and Matérn covariance function as given in Eq. (2), and $e_i$ is a residual effect with ${\mathbf {e}} \sim {\mathcal {N}}({\mathbf {0}}, {\mathbf {I}}\sigma _e^2)$. Although the data generation model (1) and this statistical model (3) are similar, we note that the statistical model is not “aware” of the 10,000 true QTL effects and the eight true spatial processes.

We modelled the genetic effect (breeding value) using a relationship matrix based either on pedigree or genome data. For the pedigree-based model, we assumed ${\mathbf {a}}\sim {\mathcal {N}}({\mathbf {0}}, {\mathbf {A}}\sigma ^2_a)$, where ${\mathbf {A}}$ is the pedigree relationship matrix [40]. We used pedigree for the phenotyped individuals (11th generation), their offspring (12th generation), and three previous generations (8–10th). For the genome-based model, we assumed ${\mathbf {a}}\sim {\mathcal {N}}({\mathbf {0}}, {\mathbf {G}}\sigma ^2_a)$, where ${\mathbf {G}}$ is the genomic relationship matrix calculated from ${\mathbf {G}} = {{\mathbf {Z}}}{{\mathbf {Z}}}^T/k$, ${\mathbf {Z}}$ was a column-centered SNP matrix, and $k = 2 \Sigma _l q_l (1-q_l)$ with $q_l$ being allele frequency of marker *l* [32].

#### Prior distributions for hyper-parameters

We used a full Bayesian analysis which requires prior distributions for all model parameters. For the intercept and fixed effects, we assumed $\sigma ^2_{\beta } = 1000$, and for the remaining variance parameters and the spatial range, we assumed penalised complexity priors [41], which are proper priors that penalise model complexity to avoid over-fitting. The penalised complexity prior for variance parameters can be specified through a quantile *u* and a probability $\alpha$ which satisfy Prob$(\sigma > u) = \alpha$, and the penalised complexity prior for the spatial range parameter through a quantile *u* and a probability $\alpha$ which satisfy Prob$(\rho < u) = \alpha$. For the variances and spatial range, we assumed penalised complexity prior distributions with quantiles *u* and probabilities $\alpha$ (Table 1).

**Table 1:** Parameters *u* and $\alpha$ for the penalised complexity priors of hyper-parameters by fitted models to the simulated and real data (see "Prior distributions for hyper-parameters" section)

Model	$u_e,\, \alpha _e$	$u_a, \, \alpha _a$	$u_h, \, \alpha _h$	$u_{s}, \, \alpha _{s}$	${u_{\rho }, \, \alpha _{\rho }}^a$	${u_{\rho }, \, \alpha _{\rho } }^b$
G	0.30, 0.50	0.10, 0.50	–	–	–	–
GH	0.15, 0.50	0.10, 0.50	0.25, 0.50	–	–	–
GS	0.15, 0.50	0.10, 0.50	–	0.25, 0.50	0.60, 0.95	50, 0.80
GHS	0.15, 0.50	0.10, 0.50	0.15, 0.50	0.10, 0.50	0.60, 0.95	50, 0.80

^a^Simulated data

^b^Real data

#### Fitted models to the simulation data

We fitted five models to the simulated data: G, GH, GS, GHS and GHSC. All models had an intercept $\beta _0$, a genetic effect $a_i$, and a residual effect $e_i$. Model GH had in addition a herd effect $h_i$, GS had in addition a spatial effect $s_i$, GHS had in addition both a herd effect and a spatial effect, and GHSC had in addition a herd effect, a spatial effect and the environmental covariates $z_i$. The models are summarised as:

$$\begin{aligned} \text {G: } y_i & =\beta _0 + a_i + e_i, \\ \text {GH: } y_i & =\beta _0 + a_i + h_i + e_i, \\ \text {GS: } y_i &=\beta _0 + a_i + s_i + e_i, \\ \text {GHS: } y_i &=\beta _0 + a_i + h_i + s_i + e_i, \\ \text {GHSC: } y_i &=\beta _0 + a_i + h_i + s_i + {\mathbf {z}}_i {\beta }_z + e_i, \\ \end{aligned}$$

where ${\mathbf {z}}_i$ is the vector of environmental covariates for individual *i* and ${\beta }_z \sim {\mathcal {N}}({\mathbf {0}}, 1000 {\mathbf {I}})$ is a vector of environmental covariate effects. The other effects were assumed distributed as described above for Eq. (3).

#### Model evaluation for simulated data

We will refer to the mean posterior genetic effect for phenotyped individuals as the estimated breeding values, and the mean posterior genetic effect for non-phenotyped individuals as the predicted breeding values. We evaluated the models using three measures: first, with the Pearson correlation (accuracy) between the true and estimated/predicted breeding values for all individuals; second, with the Spearman’s rank correlation between the true and estimated/predicted breeding values for the top 100 individuals; and third, with the continuous rank probability score (CRPS) [42], comparing the whole posterior distribution of breeding values to the true breeding values. The CRPS compares both the location and spread of the posterior distribution to the true value. The CRPS is negatively oriented, which means that lower CRPS values indicate more accurate predictions.

#### Fitted models to the real dairy cattle data

We fitted four models to the real dairy cattle data that were structurally the same as models fitted to the simulated data: G, GH, GS, and GHS. The only difference was in fixed effects that are part of the routine genetic evaluation for the analysed trait and population; an intercept $\beta _0$, three factors (year and scorer, cow’s age and stage of lactation, and year and month of calving). The genetic effect was estimated using the available pedigree. For the variances and spatial range, we assumed penalised complexity prior distributions with quantiles *u* and probabilities $\alpha$ shown in Table 1.

We used the deviance information criterion (DIC) [43] to compare the fit of the models. The DIC is widely used to compare model fit between different hierarchical Bayesian models while also assessing the model complexity. Lower values of the DIC indicate a better model fit.

### Inference

For inference, we used the Bayesian numerical approximation procedure known as the Integrated Nested Laplace Approximations (INLA) introduced by [44], with further developments described in [45, 46] and implementation available in the R-INLA package. INLA is suited for the class of latent/hierarchical Gaussian models, which includes generalised linear (mixed) models, generalised additive (mixed) models, spline smoothing methods, and models used in this study. INLA calculates marginal posterior distributions for all model parameters (fixed and random effects, and hyper-parameters) and linear combinations of effects without sampling-based methods such as Markov chain Monte Carlo (MCMC).

## Results

In this section, we present the results from fitting the models to the simulated and real data. For simulation, we compare accuracy and CRPS of estimated and predicted breeding values for the tested models. For the real data, we present posterior variances, DIC, estimated spatial effects, and how estimated breeding values differ with and without spatial modelling. All results indicate that spatial modelling improves genetic evaluation.

### Simulated data

This section presents the results from the simulation study, where the models G, GH, GS, GHS and GHSC were fitted to data with three different genetic connectedness. Overall, the results showed that in smallholder systems (i) spatial modelling increased accuracy of estimating and predicting breeding values, (ii) environmental covariates did not improve accuracy substantially beyond the distance-based spatial model, (iii) for the models without spatial effects, the accuracy of separating genetic and environmental effects was low, (iv) the benefit of spatial modelling was largest when genetic and environmental effects were strongly confounded, (v) spatial modelling in addition to the independent random herd effect did not decrease accuracy even when there was no spatial effects, and (vi) when environmental and genetic effects were confounded the accuracy improved when herds were weakly clustered rather than strongly clustered.

#### Spatial modelling increases accuracy

Spatial modelling increased accuracy of estimated and predicted breeding values. Table 2 presents the accuracy for all models and genetic connectedness scenarios. Setting the model GHSC aside for later, we observed the highest accuracy with model GHS across all scenarios. The second best was model GS, third was GH, and the worst was G. As expected genomic data improved the accuracy compared to using pedigree, and estimated breeding values were more accurate than the predicted. With weak genetic connectedness, the accuracy was low and comparable between estimation and prediction, and the pedigree models has an accuracy almost as high as the genomic models.

**Table 2:** Average accuracy of estimated breeding values (EBV) and predicted breeding values (PBV) by genetic connectedness (weak, intermediate and strong) and model with intermediate clustering of herds

	Weak		Intermediate		Strong	
	EBV	PBV	EBV	PBV	EBV	PBV
Pedigree						
G	0.33	0.28	0.32	0.18	0.32	0.20
GH	0.36	0.29	0.41	0.22	0.42	0.25
GS	0.52	0.50	0.56	0.34	0.55	0.35
GHS	0.54	0.52	0.58	0.36	0.57	0.37
GHSC	0.57	0.55	0.59	0.36	0.58	0.37
Genomic						
G	0.33	0.32	0.40	0.29	0.42	0.32
GH	0.36	0.33	0.51	0.38	0.59	0.46
GS	0.58	0.56	0.70	0.54	0.72	0.57
GHS	0.63	0.60	0.74	0.57	0.75	0.60
GHSC	0.64	0.62	0.74	0.58	0.75	0.60

Standard error for most values had an order of magnitude $10^{-3}$ with few an order of magnitude $10^{-2}$

Table 3 presents the average CRPS. The trends in the CRPS were the same as for the accuracy, with model GHS having the lowest (best) CRPS. Again, as expected genomic data improved the CRPS compared to using pedigree, and in most cases, average CRPS was lower for estimation than for prediction, but in some cases the average CRPS for prediction was slightly lower than for estimation. This improved CRPS for prediction was observed for models that did not model environmental variation and had lower accuracy (Table 2), so the lower (better) CRPS indicates that those models underestimated prediction uncertainty.

**Table 3:** Average CRPS of estimated breeding values (EBV) and predicted breeding values (PBV) by genetic connectedness (weak, intermediate and strong) and model with intermediate clustering of herds

	Weak		Intermediate		Strong	
	EBV	PBV	EBV	PBV	EBV	PBV
Pedigree						
G	0.54	0.43	0.65	0.40	0.70	0.37
GH	0.41	0.37	0.34	0.28	0.33	0.25
GS	0.17	0.17	0.17	0.18	0.18	0.18
GHS	0.16	0.16	0.17	0.18	0.18	0.18
GHSC	0.16	0.16	0.16	0.18	0.17	0.18
Genomic						
G	0.39	0.39	0.32	0.30	0.30	0.26
GH	0.36	0.37	0.22	0.22	0.18	0.18
GS	0.15	0.15	0.13	0.15	0.13	0.15
GHS	0.14	0.15	0.12	0.15	0.12	0.14
GHSC	0.14	0.14	0.12	0.15	0.12	0.14

Standard error for all values had an order of magnitude $10^{-3}$

The rank correlations for the top 100 individuals were in line with accuracy (Table 2) and CRPS (Table 3) results for all individuals. We show this in Additional file 3: Table S1. These results show that spatial modelling (models GS, GHS and GHSC) improved accuracy of ranking the top individuals compared to no spatial modelling (models G and GH).

#### Including environmental covariates

The environmental covariates did not improve the results substantially beyond the simple distance-based relationships between herds. This is shown for accuracy in Table 2 and CRPS in Table 3. The accuracy and CRPS were only marginally better for the GHSC model compared to the GHS model in some cases, and in the remaining cases, they were comparable. Because of this, we focused on the sufficient models and excluded model GHSC in the remaining results. Some additional results with model GHSC are given in Additional file 3.

#### Separating genetic and spatial (environmental) effects

The models without spatial effects were not able to accurately separate genetic and spatial (environmental) effects. In Table 4, we present the correlations between the estimated breeding values and the true spatial effects by model and genetic connectedness. Models G and GH had a high correlation, which suggests that estimated breeding values captured parts of the spatial effects. Models GS and GHS had correlations closer to zero, which suggests that these models separated genetic and spatial effects more accurately. This, together with the correlation results in Table 2 and CRPS results in Table 3, suggests that the herd effect alone is not sufficient to account for all environmental effects in smallholder systems.

**Table 4:** Average correlation between estimated breeding values and true spatial effect by genetic connectedness (weak, intermediate and strong) and model

	Weak	Intermediate	Strong
Pedigree			
G	0.68	0.64	0.64
GH	0.70	0.60	0.58
GS	0.11	0.06	0.06
GHS	0.12	0.06	0.06
Genomic			
G	0.84	0.74	0.69
GH	0.83	0.63	0.50
GS	0.16	0.05	0.04
GHS	0.21	0.05	0.04

Standard error for all values had an order of magnitude $10^{-3}$

#### Comparing genetic connectedness scenarios and genetic models

The benefit of spatial modelling was largest when spatial and genetic effects were difficult to separate. In Additional file 2: Figure S1, we show the relative improvement in accuracy and CRPS between models GH and GHS by genetic connectedness. With both the genome and pedigree data, the improvement was largest with weak genetic connectedness (about 50% to 80%), second with intermediate genetic connectedness (about 35% to 65%), and third with strong genetic connectedness (about 20% to 45%). These settings range between strongly confounded genetic and spatial effects, to separable genetic and spatial effects. With weak genetic connectedness, there was little difference in improvement between models using genomic or pedigree data, whereas with intermediate and strong genetic connectedness there was a tendency for the improvement to be largest with the pedigree data.

#### Changing proportion of spatial variance

**Fig. 2:**
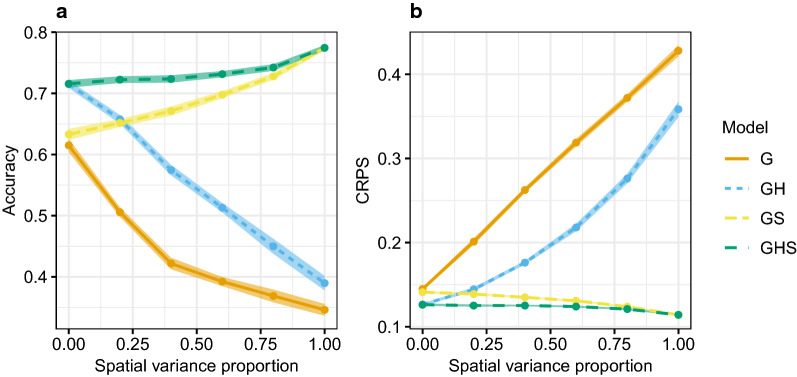
Average accuracy (**a**) and CRPS (smaller is better) (**b**) with 95% confidence intervals for estimated breeding values by proportion of spatial variance in the sum of spatial and herd variance in the scenario with intermediate genetic connectedness and using the genomic model

Spatial modelling, in addition to an independent random herd effect even when there were no spatial effects, did not decrease the accuracy. In Fig. 2, we present the accuracy and CRPS for estimated breeding values when using genomic data under intermediate genetic connectedness. The *x*-axis goes from all environmental variance covered by herd effects to all covered by spatial effects. For models G and GH, the accuracy and CRPS worsened as the proportion of spatial variance increased, whereas for models GS and GHS the accuracy and CRPS improved. Overall, model GHS had the highest accuracy and lowest (best) CRPS for all spatial variance proportions. It was as good as model GH when there was no spatial variation and as model GS when there was no herd effect variation.

From the results so far, we have seen that model GS had better accuracy and CRPS than model GH. However, this is not always the case. When most of the environmental variation was due to herd effects rather than spatial effects, model GH gave better estimates than model GS.

The same tendencies were seen for the predicted breeding values for both genomic and pedigree-based models, and in other genetic connectedness scenarios, as shown in the tables presented in Additional file 3.

#### Changing the herd clustering

**Fig. 3:**
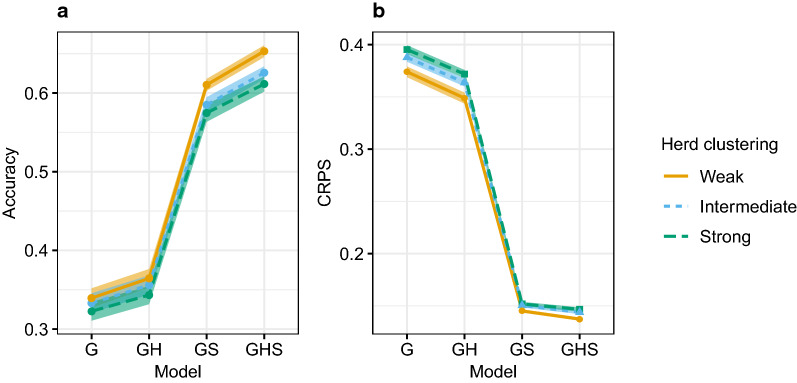
Average accuracy (**a**) and CRPS (smaller is better) (**b**) with 95% confidence intervals by model and herd clustering in the scenario with weak genetic connectedness and using the genomic model

When spatial and genetic effects were confounded, the accuracy of estimation improved when herds were weakly clustered rather than strongly clustered. When simulating the data, we varied the distribution of herd locations, from strongly clustered to less clustered around each village centre. In Fig. 3, we present the accuracy and CRPS for estimated breeding values using genomic data under weak genetic connectedness for the three clustering levels. Figure 3 shows that as herds were less clustered, the accuracy and CRPS improved across all models. We observed the same trend for predicted breeding values and using pedigree data, but not with intermediate and strong genetic connectedness, where the genetic and spatial effects were less confounded. Tables showing the accuracy and CRPS between true and inferred breeding values and the correlation between inferred breeding values and the true spatial effects for all levels of genetic connectedness and herd clustering are in Additional file 3.

### Real data

In this section, we present the results from fitting the models to the subset of real dairy cattle data. We present the posterior distributions of the hyper-parameters, the DIC, the estimated spatial field from model GHS, and compare the estimated breeding values from models GH and GHS. The corresponding results for the full data set are in Additional file 2 and Additional file 3: Table S15. Overall, the results showed that (i) models GH and GHS explained most of the variation in the data and had the best fit, (ii) the data had a spatially dependent structure captured by models GS and GHS, and (iii) the two models with the best fit, GH and GHS, separated the genetic and environmental effects differently for animals living in areas with relatively large spatial effects.

#### Explained variation and model fit

**Fig. 4:**
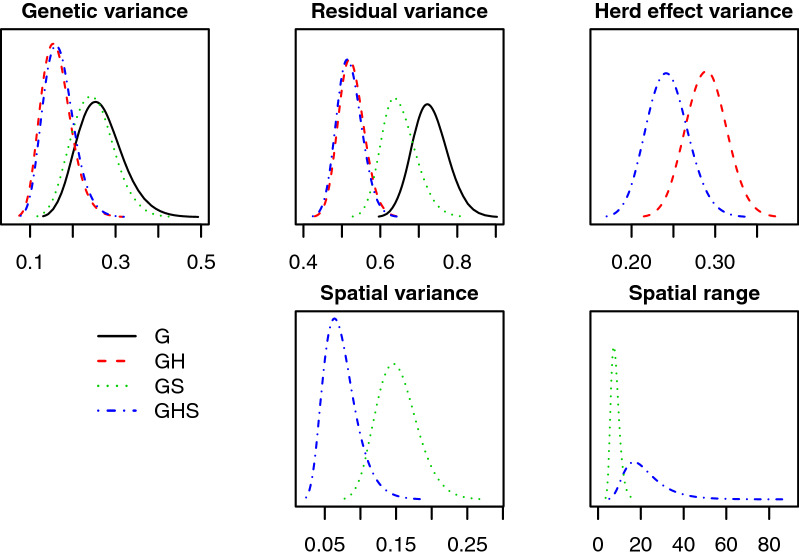
Posterior distributions of hyper-parameters from models G, GH, GS and GHS fitted to the real data

Models GH and GHS explained most of the variation in the data and had the best fit according to DIC. In Fig. 4, we show the posterior distributions for the model hyper-parameters. Figure 4 has five panels showing additive genetic variance $\sigma _a^2$, residual variance $\sigma _e^2$, herd effect variance $\sigma _h^2$, spatial variance $\sigma _s^2$, and spatial range $\rho$ in km.

The posterior additive genetic variance was similar between models GH and GHS, larger in model GS, and even larger in model G. The same tendency was seen for the posterior residual variance. The posterior herd effect variance was smaller in model GHS than in model GH, which was reasonable since the herd effect in model GH captured the spatial component of the phenotype, which model GHS assigned to the spatial effect. The posterior spatial variance in model GS was larger than in model GHS since model GS captured herd effects. Finally, the posterior spatial range was smaller in model GS than in model GHS, since model GS captured herd effects in the spatial effects, which means shorter range of dependency between spatial locations. The mean posterior range from model GHS indicated that herds more than 22 km apart had close to independent (large scale) environments.

Since model G cannot separate variation due to herd or other environmental effects, it is possible that some of the estimated genetic effects were confounded with other effects, which explains the high estimate found for the additive genetic variance with this model. A similar reasoning could be used for model GS, which assigned variation due to herd effects, either to genetic, spatial or residual effects. From Fig. 4 it seems that the variation from herd effects was distributed to all other effects, which explains why the estimated additive genetic variance and estimated residual variance were larger in model GS than in models GH and GHS, and why the estimated spatial variance was larger than in model GHS. It seems that models GH and GHS distributed variation similarly except for the herd effect, which is expected to be higher in model GH than in model GHS.

Table 5 shows the DIC for each model and indicates that model GHS had the best fit, followed by model GH, then model GS and finally model G. These numbers are in line with the estimated hyper-parameters, that showed that models GHS and GH could explain most of the variation in the phenotype. Although model GS also has the potential to explain much of the variation, it is forced to assign herd effects either to genetic or spatial effects. We saw from the results with the simulated data that model GS had a worse model fit than model GH when most of the environmental variation was due to herd effects, which seems to be the case here considering the small posterior spatial variance. Finally, model G was not able to separate genetic and environmental effects, which leads to a poor model fit. A rule of thumb is that a complex model should be preferred over a less complex model if the DIC is reduced by more than ten units. When it comes to choosing between models GH and GHS, model GHS should be preferred, as its DIC was 36 units smaller.

**Table 5:** Deviance information criterion (DIC) by model fitted to the real data

Model	DIC
G	10,494
GH	9795
GS	10,233
GHS	9759

#### The estimated spatial effects

**Fig. 5:**
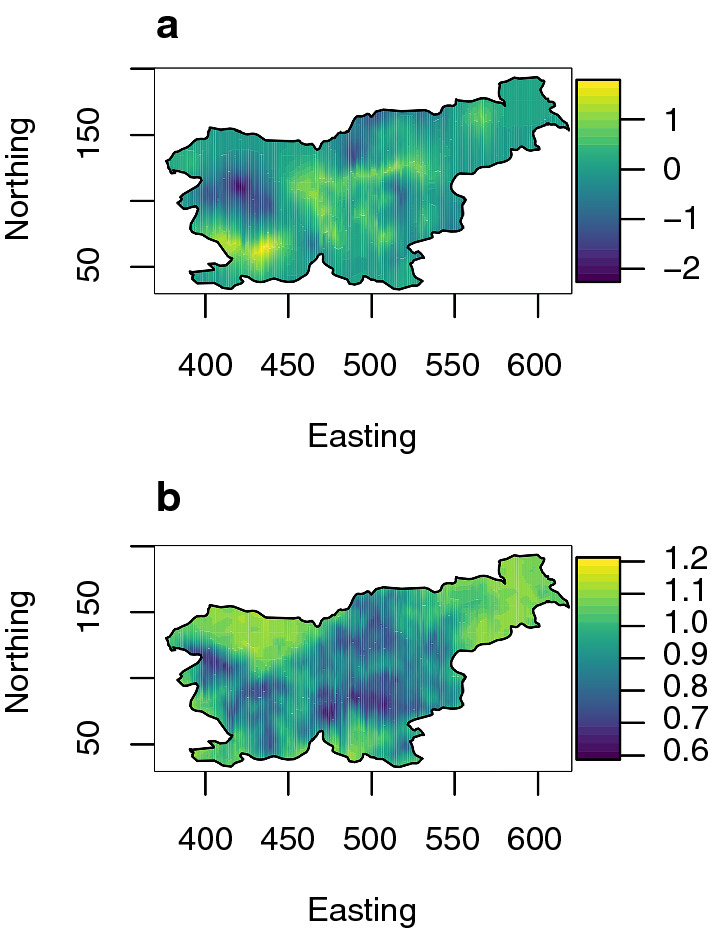
Posterior mean (**a**) and standard deviation (**b**) of the estimated spatial effect (in units of posterior spatial standard deviation) from model GHS fitted to the real data—the axis units are in km

The data had a spatially dependent structure captured by models GS and GHS, and the estimated spatial field from model GHS is shown in Fig. 5. Figure 5 shows the estimated mean (posterior mean), in panel (a), and uncertainty (posterior standard deviation) in panel (b). The axes show coordinates in the Transverse Mercator coordinate system in km using datum WGS84.

In the western part of Slovenia, model GHS suggests two environmental regions with a mean different from zero, one with a positive effect, and one with a negative effect. In the central part of Slovenia, there are several smaller regions with either a positive or negative effect. In the northeast part of Slovenia, there were not many observations, so there is only a small region with a positive effect, and zero effects otherwise. These estimates are in line with the natural geographic conditions in Slovenia. The magnitude of these spatial effects ranges from $-\,2.2$ to 1.7 posterior spatial standard deviations. The uncertainty was lowest where observations were available and was highest where there were no observations.

#### Comparing breeding values from models GH and GHS

The two models with the best fit, models GH and GHS, separated the genetic and environmental effects differently for animals living in areas with relatively large spatial effects.

The DIC in Table 5 and the estimated hyper-parameters in Fig. 4, indicated that models GH and GHS had the best model fit and a similar decomposition of the genetic and environmental variation. Furthermore, the estimated breeding values from models GH and GHS were highly correlated, with a correlation of about 0.995.

To evaluate how well models separated genetic and environmental effects, we computed the correlation between estimated breeding values from models GH and GHS with estimated spatial effects from model GHS. For model GH, this correlation was about 0.14, whereas for model GHS it was about 0.07. This suggests that there were some effects that were assigned as genetic effects in model GH, but assigned as spatial effects in model GHS.

**Fig. 6:**
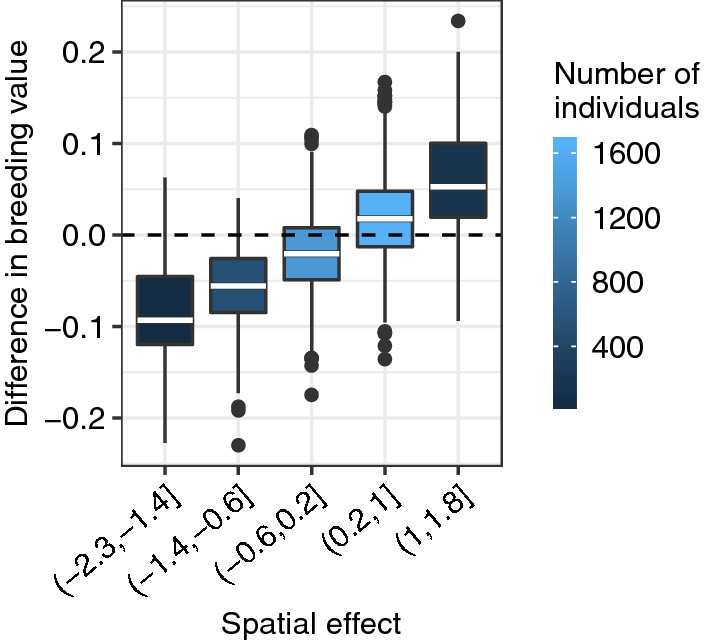
The difference in estimated breeding values (in units of posterior genetic standard deviation) between models GH and GHS by the estimated spatial effect (in units of posterior spatial standard deviation) from model GHS fitted to the real data

Figure 6, presents the differences in estimated breeding values between models GH and GHS as boxplots according to estimated spatial effects from model GHS. This shows that the difference was correlated with spatial effect from model GHS. When estimated spatial effects were negative, estimated breeding values from model GH were smaller than from model GHS. When estimated spatial effects were positive, estimated breeding values from model GH were larger than from model GHS. The magnitude of the difference ranged from $-\,0.2$ to 0.2 posterior genetic standard deviations, which indicates confounding for animals living in areas with large spatial effects. The figure also shows how many cows were used in each boxplot, which indicates that, for a majority of the cows, the difference in estimated breeding values was not large.

The correlation between differences in estimated breeding values and estimated spatial effects from model GHS was about 0.62. This is in line with what we saw from the simulation results, and suggests that although the two models had highly correlated estimated breeding values, there were differences between estimated breeding values for animals in regions with large spatial effects.

We also compared the top 10 and 20 ranked cows and bulls from the models GH and GHS, to see if a difference in estimated breeding value influenced ranking. We found that the difference was not critical for ranking since the top cows and bulls were present in areas with relatively small spatial effects. For the cows, we had an overlap of 7 (18) cows when comparing the top 10 (20) from each model. For the bulls, we had an overlap of 9 (18) bulls when comparing the top 10 (20).

## Discussion

The results show that spatial modelling improves genetic evaluation in smallholder systems. In particular, it increases the accuracy of genetic evaluation under weak genetic connectedness by establishing environmental connectedness, and with this, more accurate separation of genetic and environmental effects. These observations highlight two broad points for discussion: (i) why does spatial modelling improve genetic evaluation and (ii) what are the limitations of this study and future possibilities.

### Why spatial modelling improves genetic evaluation

Spatial modelling improves genetic evaluation because it separates environmental variation that is common to nearby herds more accurately from the other effects on the phenotype. Since spatial effects are estimated jointly for all herds and other effects, this induces environmental connectedness and, in turn, enhances separation of environmental and genetic effects. Animal breeders are very aware of the data structure that is required for accurate genetic evaluation [7–9] and there are formal methods to assess genetic connectedness between contemporary groups [14, 15, 47–49]. An interesting future work would be to extend these methods to account for environmental connectedness. Achieving sufficient genetic connectedness is particularly difficult when contemporary groups are small and there is limited genetic connectedness between them.

A way to increase genetic connectedness is to use genomic data, although this was not sufficient in our case. Using genomic data reveals more genetic connectedness than pedigree data because animals likely share at least some alleles, and this has been shown to increase the accuracy of genetic evaluation [7, 50, 51]. However, our targeted setting consisted of smallholder herds, which are an extreme case of challenging data structure for genetic evaluation. Furthermore, we varied genetic connectedness between herds and villages. We found that across all genetic connectedness scenarios, spatial modelling increased accuracy more than using genomic data instead of pedigree data. Furthermore, with the weakest genetic connectedness, genomic data was not effective at all, while spatial modelling was. This is, in a way, not surprising because our herds were so small that we had strong confounding between genetic and environmental effects, as well as weak genetic connectedness. Genomic data could not separate genetic and environmental effects, since herds were too small for accurate estimation of their effect, even with random effects. In this case, spatial modelling, at least environmentally, connected nearby herds and created effective contemporary groups. These results show that in addition to genomics other tools are also needed to improve smallholder systems [52]. As expected accuracy was low in this extreme setting, although surprisingly not very low (see the next sub-section on possible reasons). These scenarios might seem too extreme, but they are a reflection of real situations in many countries around the world, e.g. [11].

Spatial modelling has a long tradition and has already been used in animal breeding, e.g. [23, 53]. We have used it in the extreme scenario of small herds and for this reason we used the geostatistical approach that accounts for the fine-grained herd coordinate information. An alternative approach could be to cluster herds into village groups and possibly further cluster villages into region groups. In this case, we could model the village groups as an independent fixed or random effect to account for small scale environmental (management) effects, and possibly further model the region groups as a dependent random effect accounting for covariance between neighbouring regions to account for large-scale environmental effects [24, 25]. An issue with this approach is that we lose the ability to model each individual herd, and that administrative regions often do not represent correctly geography and other environmental effects. Given that the clustering approach has trade-offs, that there are efficient geostatistical models that adapt to data, and that efficient and easy to use implementations exist, we recommend the use of geostatistical models.

We recommend routine use of spatial modelling in quantitative genetic models. Namely, collected data will always come from some area with likely variation in environmental effects. Our results show that spatial modelling is robust even when there is no spatial variation. The observed gains from this study will likely be smaller in cases with larger herds, but even in those cases, spatial modelling can induce environmental connectedness, and it can also provide estimates of spatial effects. These estimates could be used to target interventions or policies. Importantly, our analysis of simulated and real data indicates that spatial modelling can separate environmental and genetic effects more accurately. Such modelling improvements will also be very useful beyond animal breeding populations; for example, in quantitative genetic analyses of human populations and wild populations. These populations also have similarly challenging data structure with rampant population structure (genetic disconnectedness) [54, 55] and the existence of biases in estimated genetic effects in line with geographic variation has been reported [56].

In line with the potential of spatial modelling to account for spatial variation, we recommend a geographically broad collection of data to train robust models. Genomics is revolutionising breeding in developed and developing countries [6, 7, 32]. To deliver its full potential, breeding organisations should ensure broad geographic coverage when collecting data. This will avoid bias towards a specific region, in particular with genomic prediction. Spatial modelling can account for variation between and within regions, but it needs data from the regions to estimate optimal model parameters.

In relation to data collection guidance, we were surprised to find that environmental covariates did not improve the accuracy of genetic evaluation beyond simple distance-based relationships between herds. Here, we simulated the total spatial effect as a sum of eight spatial processes with a range of model parameters that made the processes quite different and we assumed that we could observe these with some noise. Our hypothesis was that modelling the observed environmental covariates would reveal the underlying spatial processes and increase accuracy in the same way that the use of genomic data reveals the underlying genetic process behind the pedigree expectations [32, 33]. There are at least three possible explanations for this. First, we simulated a small number of spatial processes, and the distance-based relationships were sufficient to model spatial variation. Second, the noise in observations was larger than the signal or our data set was too small to capture the signal. Third, the two-dimensional form of the space constrains the value of environmental covariates for increasing accuracy beyond the distance-based relationships. More studies are needed to address this question.

### The limitations of this study and future possibilities

There is a huge number of possible scenarios and parameter combinations that we could have tested. For example, we assumed the absence of non-additive genetic effects, genotype-by-environment interaction, data errors, heterogeneous variances and considered only a single trait and breed. Furthermore, the animals were initially distributed to herds randomly, and the farms using artificial insemination were chosen randomly. Such simplifications are likely to yield higher accuracies than expected in real smallholder systems. However, the analysis of real data corroborates the main conclusions from the simulations. Future studies could, for example, consider non-random distribution of animals among herds as well as the use of artificial insemination and the best bulls. These non-random associations are real since well-resourced farmers are more likely to use artificial insemination and the best bulls [22]. With the real data analysis, we tried to mimic a smallholder setting by using only a subset of the data. However, it should be noted that this data has a much higher level of artificial insemination than most smallholder systems, even in the strong genetic connectedness scenario in our simulation.

Genotype-by-environment interactions have been modelled in several studies [53, 57–60] and such interactions are likely to be substantial in smallholder systems, in particular when native and exotic breeds are used [6]. We ignored these interactions in our study. Of particular notice regarding these interactions and in relation to our work is the study of [53]. They used geographical location and weather data in addition to herd summaries to describe environmental conditions in genetic evaluations, with and without genotype-by-environment interactions and concluded that the farming environment explained variation in the data, as well as the genotype-by-environment component. Further work is needed to embrace the rich set of tools from the spatial statistics community to address genotype-by-environment interactions [61, 62].

Yet another important source of phenotypic variation that we ignored are heterogeneous variances, which are also likely to be substantial in smallholder systems. There are multiple models and methods used by breeders and geneticists to account for such variation, e.g. [63–65]. We note that there is also a rich spatial literature on models that can deal with non-stationarity in dependency and variance, e.g. [29, 30, 66–68], which for example could enable the modelling of directional dependence based on local anisotropy, e.g. [69]. Using and benefitting from non-stationary models can be challenging due to computational costs and the amount of data needed to fit these models [70]. However, this will become increasingly possible and desired as data sets increase in size with the progression of the digital revolution in agriculture and more computationally efficient methods become available.

Breeding programmes interested in spatial modelling will have to invest in software modification. This is not a limitation of this study, but interested breeding programmes would either have to use the R-INLA package [44] or implement an extension of their existing software. While the R-INLA package is a mature project, it does not support all animal breeding models, most notably multi-trait models. However, it handles a rich set of likelihoods (Gaussian, Poisson, Bernoulli, Weibull, etc.), link functions, independent or correlated random effects (time-series, regions, points, generic such as pedigree, etc.) and priors. It uses the same key underlying linear algebra routines as standard genetic evaluation software [25, 71–73], and enables both full Bayesian analysis with fast and very accurate approximate algorithm [74] or even faster empirical Bayesian analysis. We have used the R-INLA package extensively for standard quantitative genetic studies [75–77], accounting for selection [78], spatial modelling of plant and tree trials [79] and for modelling of phenotypes on phylogeny [80]. While the R-INLA package is fast for models with a sparse structure (time-series, spatial regions or points and pedigree), it does not fare well for genomic models that have dense a structure [32, 33]. However, use of recently proposed approximate genomic models [34, 35] and sparse-dense libraries would help [81, 82]. A simple alternative for spatial modelling with standard software such as [83, 84], would be to force the setup and inversion of the spatial covariance matrix using Gaussian model. This would suffice for a few thousand well-dispersed herds, but might lead to numeric issues with nearby herds (near matrix singularity) or much larger numbers of herds that will soon become a reality with the digital revolution of agriculture.

Furthermore, since INLA does a full Bayesian analysis, the user has to set prior distributions for all model parameters. This is not always straightforward, but setting a prior based on the knowledge about the process is likely to improve inference substantially, particularly when data is sparse. There is a number of ways to set mildly informative priors. We used penalised complexity priors [41] since these avoid over-fitting and can accommodate prior knowledge about the relative importance of different effects [85, 86].

## Conclusions

The take-home message from this study is that spatial modelling can improve genetic evaluation in smallholder systems by inducing environmental connectedness, and with this can enhance separation of genetic and environmental effects beyond an independent herd effect. We have demonstrated this with simulated data with different levels of genetic connectedness, proportions of spatial to management (herd) variation, herd clustering and pedigree or genomic modelling. These results have to be further corroborated with a range of smallholder datasets for which we also have to account for multiple breeds and their crosses, genotype-by-environment interactions and heterogeneous variances. We expected that environmental covariates would improve spatial modelling following the analogy of genetic modelling with observed genomic versus expected pedigree data, but this was not the case in our simulations. Based on all these results, we suggest routine spatial modelling in genetic evaluations, particularly for smallholder systems. Spatial modelling could also have a major impact in studies of human and wild populations.

## Supplementary information

**Additional file 1.** Simulation code available from https://doi.org/10.6084/m9.figshare.12403898.

**Additional file 2.** Additional figures.

**Additional file 3.** Additional tables.

## Data Availability

The scripts for data simulation and model fitting are available in Additional file 1. The real data are owned by the Slovenian Brown-Swiss breeding programme and were prepared for this study by Jana Obšteter (Agricultural Institute of Slovenia) and Barbara Luštrek (University of Ljubljana).
