# Supplementary material for: Spatial modelling improves genetic evaluation in smallholder breeding programs
Source: Genet Sel Evol. 2020 Nov 16;52:69. doi: 10.1186/s12711-020-00588-w (PMC7670695; doi:10.1186/s12711-020-00588-w)
Supplement: Supplementary file 2 — Additional file 2. Additional figures. [file 12711_2020_588_MOESM2_ESM.pdf]

Additional figures: Spatial modelling improves genetic evaluation in smallholder breeding programs

Maria L. Selle, Ingelin Steinsland, Owen Powell, John M. Hickey, Gregor Gorjanc

November 1, 2020

Simulated data

Comparing genetic connectedness and genetic models

In Figure S1 we plot the relative improvement in average accuracy and CRPS between true breeding values (TBV) and estimated breeding values (EBV) or predicted breeding values (PBV) when using models GH or GHS, for the different levels of genetic connectedness.

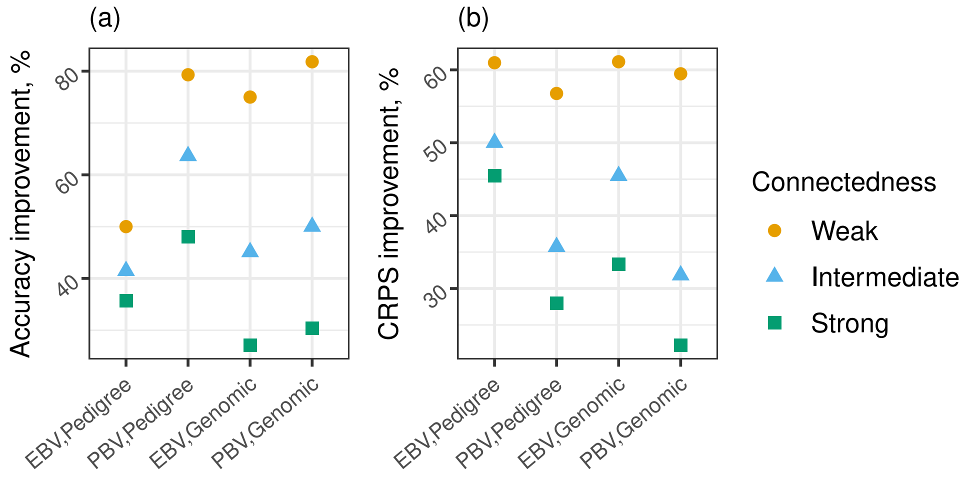

Figure S1: Percentage improvement in EBV accuracy (a) and CRPS (b) between models GH and GHS by genetic connectedness and genetic model

Real data

The herd locations of the 1,838 different herds in Slovenia are shown in Figure S2. The axes show the coordinates in kilometres in the Transverse Mercator coordinate system using datum WGS84.

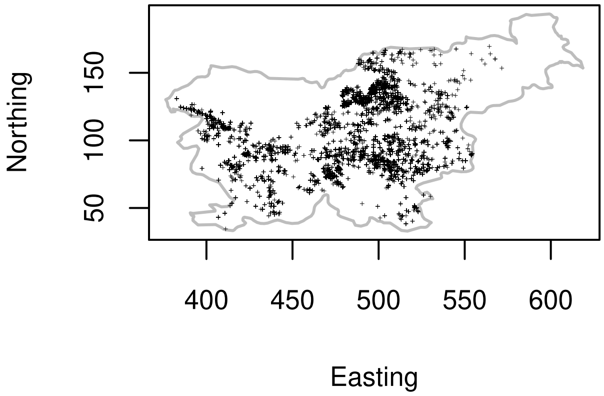

Figure S2: The location of the herds in the BSC data shown with black points, and the border of Slovenia in grey. The axis units are in km

For the models G, GH, GS and GHS applied to the full real data, we present the posterior hyper-parameters in Figure S3, the posterior mean and standard deviation of the estimated spatial effects from model GHS in Figure S4, and the difference in EBV between models GH and GHS plotted against the mean posterior spatial effect from model GHS in Figure S5.

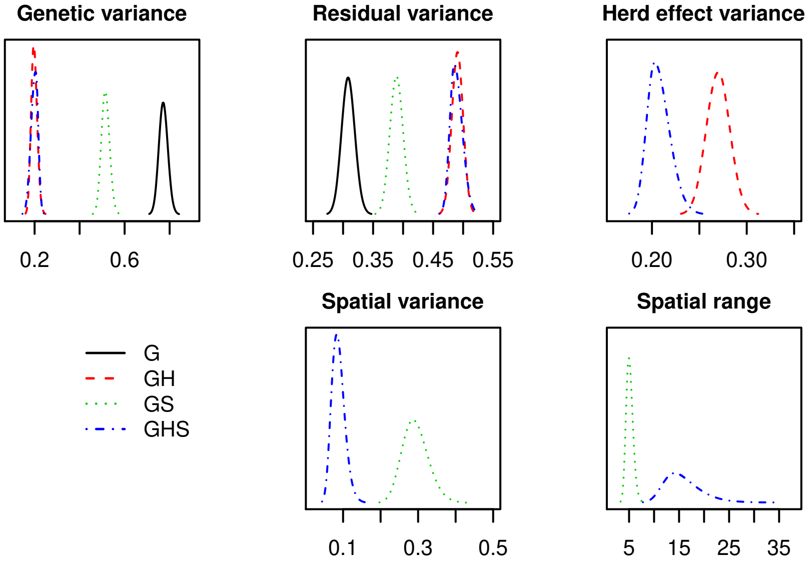

Figure S3: Posterior distributions of hyper-parameters from models G, GH, GS and GHS applied to the full real data

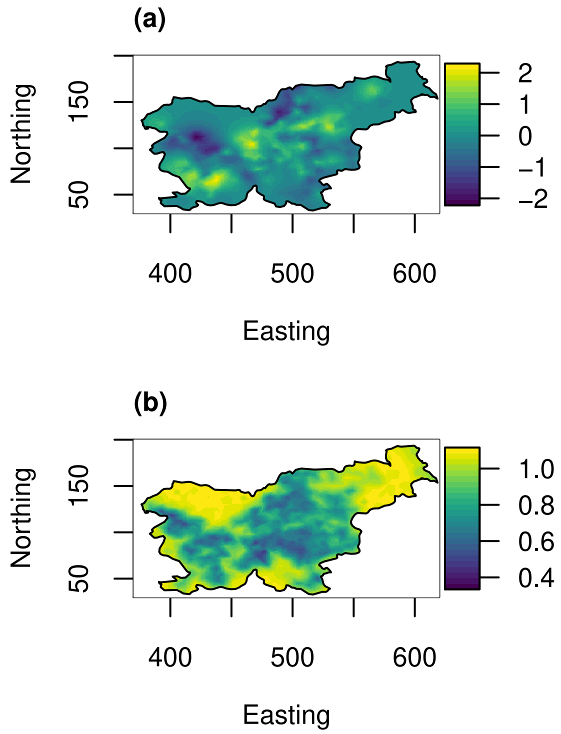

Figure S4: Posterior mean (a) and standard deviation (b) of the estimated spatial effect (in units of spatial standard deviation) from model GHS fitted to the real data - the axis units are in km

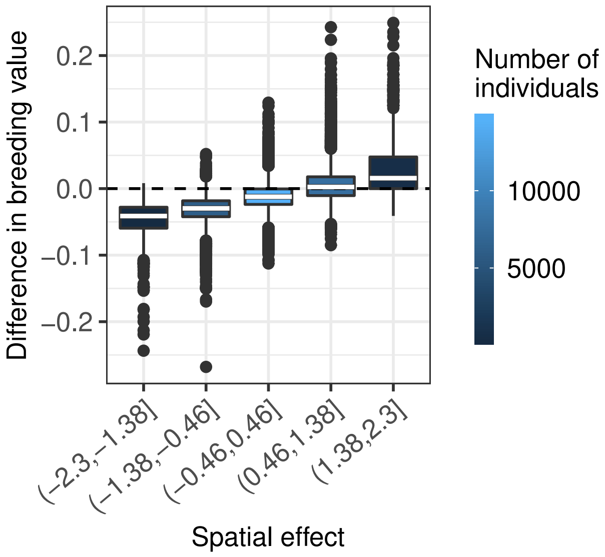

Figure S5: The difference in estimated breeding values (in units of genetic standard deviation) between models GH and GHS by the estimated spatial effect (in units of spatial standard deviation)
