# Supplementary material for: Spatial modelling improves genetic evaluation in smallholder breeding programs
Source: Genet Sel Evol. 2020 Nov 16;52:69. doi: 10.1186/s12711-020-00588-w (PMC7670695; doi:10.1186/s12711-020-00588-w)
Supplement: Supplementary file 3 — Additional file 3. Additional tables. [file 12711_2020_588_MOESM3_ESM.pdf]

Additional tables: Spatial modelling improves genetic evaluation in smallholder breeding programs

Maria L. Selle, Ingelin Steinsland, Owen Powell, John M. Hickey, Gregor Gorjanc

November 1, 2020

Simulated data

Rank correlations

Table S1 shows the average rank correlation for the top 100 individuals between the true and estimated or predicted breeding values for all levels of genetic connectedness, using pedigree or genomic data, when the herd locations were simulated from a bi-variate normal distribution with mean equal to the village locations, and variance $3.5 \cdot 10^{-4}\mathbf{I}_{2\times 2}$ (intermediate herd clustering).

Table S1: Average rank correlation for the top 100 individuals over 60 replications for the different levels of genetic connectedness, using pedigree or genomic markers, and for both estimated breeding values (EBV) and predicted breeding values (PBV). The standard error had order of magnitude 10^{-2}.

	Weak		Intermediate		Strong	
	EBV	PBV	EBV	PBV	EBV	PBV
**Pedigree**						
G	0.11	0.09	0.09	0.05	0.06	0.05
GH	0.12	0.09	0.11	0.07	0.08	0.07
GS	0.18	0.20	0.14	0.07	0.12	0.11
GHS	0.19	0.20	0.16	0.08	0.13	0.11
GHSC	0.21	0.20	0.16	0.08	0.13	0.11
**Genomic**						
G	0.12	0.20	0.11	0.17	0.11	0.18
GH	0.13	0.21	0.15	0.23	0.16	0.26
GS	0.24	0.37	0.20	0.32	0.24	0.35
GHS	0.26	0.39	0.23	0.35	0.25	0.37
GHSC	0.25	0.41	0.23	0.36	0.25	0.37

Changing proportion of spatial variance

Here we show the average accuracy and CRPS between TBV and EBV or PBV for all levels of genetic connectedness, using both pedigree and genomic data, when varying the proportion of spatial variance relative to the sum of spatial variance and herd effect variance. The herd locations were simulated from a bi-variate normal distribution with mean equal to the village locations, and variance $3.5 \cdot 10^{-4}\mathbf{I}_{2\times 2}$ (intermediate herd clustering).

Table S2 and Table S3 respectively show accuracy and CRPS for weak genetic connectedness. Table S4 and Table S5 respectively show accuracy and CRPS for intermediate genetic connectedness. Table S6 and Table S7 respectively show accuracy and CRPS for strong genetic connectedness.

Table S2: Average accuracy for EBV and PBV with weak genetic connectedness, using pedigree or genomic data, with varying proportion of spatial variance. The standard error for some values had order of magnitude 10^{-2}, and most had 10^{-3}

	EBV						PBV					
$\sigma_s^2/(\sigma_s^2+\sigma_h^2)$	0	0.2	0.4	0.6	0.8	1	0	0.2	0.4	0.6	0.8	1
**Pedigree**												
G	0.53	0.40	0.35	0.34	0.32	0.31	0.54	0.38	0.32	0.29	0.26	0.23
GH	0.57	0.46	0.39	0.37	0.34	0.32	0.57	0.42	0.34	0.31	0.27	0.24
GS	0.51	0.48	0.49	0.52	0.56	0.60	0.51	0.46	0.47	0.50	0.53	0.58
GHS	0.57	0.53	0.53	0.54	0.56	0.60	0.56	0.51	0.51	0.52	0.53	0.58
**Genomic**												
G	0.54	0.40	0.36	0.34	0.31	0.32	0.55	0.38	0.34	0.31	0.27	0.30
GH	0.64	0.47	0.40	0.36	0.33	0.33	0.62	0.43	0.37	0.33	0.28	0.30
GS	0.53	0.51	0.54	0.57	0.63	0.70	0.53	0.50	0.53	0.55	0.60	0.67
GHS	0.64	0.60	0.61	0.62	0.64	0.70	0.62	0.58	0.58	0.58	0.61	0.67

Table S3: Average CRPS for EBV and PBV with weak genetic connectedness, using pedigree or genomic data, with varying proportion of spatial variance. The standard error for all values had order of magnitude 10^{-3}

	EBV						PBV					
$\sigma_s^2/(\sigma_s^2+\sigma_h^2)$	0	0.2	0.4	0.6	0.8	1	0	0.2	0.4	0.6	0.8	1
**Pedigree**												
G	0.164	0.271	0.395	0.552	0.688	0.730	0.164	0.255	0.347	0.429	0.489	0.542
GH	0.155	0.237	0.332	0.406	0.492	0.618	0.155	0.213	0.289	0.359	0.431	0.519
GS	0.161	0.170	0.171	0.170	0.168	0.165	0.162	0.155	0.155	0.152	0.149	0.142
GHS	0.155	0.164	0.167	0.166	0.166	0.164	0.155	0.146	0.148	0.147	0.147	0.142
**Genomic**												
G	0.158	0.242	0.317	0.385	0.452	0.514	0.158	0.237	0.313	0.384	0.450	0.524
GH	0.139	0.214	0.287	0.359	0.436	0.508	0.142	0.209	0.284	0.357	0.433	0.517
GS	0.154	0.160	0.157	0.153	0.144	0.129	0.155	0.141	0.142	0.138	0.131	0.116
GHS	0.139	0.148	0.147	0.146	0.140	0.129	0.142	0.134	0.135	0.133	0.127	0.116

Table S4: Average accuracy for EBV and PBV with intermediate genetic connectedness, using pedigree or genomic data, with varying proportion of spatial variance. The standard error for some values had order of magnitude 10^{-2}, and most had 10^{-3}

	EBV						PBV					
$\sigma_s^2/(\sigma_s^2+\sigma_h^2)$	0	0.2	0.4	0.6	0.8	1	0	0.2	0.4	0.6	0.8	1
**Pedigree**												
G	0.51	0.40	0.35	0.33	0.32	0.32	0.29	0.22	0.18	0.17	0.17	0.16
GH	0.61	0.51	0.44	0.39	0.36	0.33	0.36	0.29	0.24	0.21	0.18	0.16
GS	0.53	0.53	0.55	0.59	0.61	0.65	0.31	0.32	0.33	0.35	0.37	0.41
GHS	0.61	0.60	0.60	0.62	0.62	0.65	0.37	0.37	0.37	0.38	0.38	0.41
**Genomic**												
G	0.61	0.51	0.42	0.39	0.37	0.35	0.46	0.40	0.33	0.26	0.26	0.26
GH	0.72	0.66	0.57	0.51	0.45	0.39	0.56	0.52	0.44	0.36	0.33	0.28
GS	0.63	0.65	0.67	0.70	0.73	0.77	0.47	0.52	0.54	0.54	0.57	0.60
GHS	0.72	0.72	0.72	0.73	0.74	0.77	0.56	0.58	0.58	0.57	0.58	0.60

Table S5: Average CRPS for EBV and PBV with intermediate genetic connectedness, using pedigree or genomic data, with varying proportion of spatial variance. The standard error for all values had order of magnitude 10^{-3}

	EBV						PBV					
$\sigma_s^2/(\sigma_s^2+\sigma_h^2)$	0	0.2	0.4	0.6	0.8	1	0	0.2	0.4	0.6	0.8	1
**Pedigree**												
G	0.165	0.268	0.396	0.536	0.685	0.709	0.181	0.233	0.306	0.364	0.411	0.431
GH	0.153	0.211	0.296	0.370	0.455	0.578	0.174	0.189	0.239	0.284	0.335	0.398
GS	0.160	0.167	0.164	0.159	0.159	0.153	0.178	0.160	0.158	0.156	0.154	0.150
GHS	0.153	0.161	0.159	0.157	0.158	0.153	0.173	0.156	0.154	0.154	0.153	0.150
**Genomic**												
G	0.145	0.201	0.262	0.319	0.372	0.428	0.162	0.187	0.235	0.285	0.322	0.364
GH	0.126	0.144	0.176	0.218	0.276	0.358	0.148	0.141	0.165	0.203	0.244	0.311
GS	0.141	0.139	0.135	0.131	0.124	0.114	0.159	0.134	0.129	0.130	0.125	0.120
GHS	0.126	0.125	0.125	0.124	0.121	0.114	0.148	0.125	0.123	0.125	0.122	0.120

Table S6: Average accuracy for EBV and PBV with strong genetic connectedness, using pedigree or genomic data, with varying proportion of spatial variance. The standard error for some values had order of magnitude 10^{-2}, and most had 10^{-3}

	EBV						PBV					
$\sigma_s^2/(\sigma_s^2+\sigma_h^2)$	0	0.2	0.4	0.6	0.8	1	0	0.2	0.4	0.6	0.8	1
**Pedigree**												
G	0.49	0.38	0.32	0.32	0.31	0.32	0.30	0.24	0.20	0.19	0.18	0.17
GH	0.57	0.50	0.44	0.41	0.38	0.35	0.36	0.32	0.27	0.25	0.22	0.19
GS	0.51	0.52	0.53	0.55	0.57	0.61	0.31	0.33	0.34	0.35	0.36	0.39
GHS	0.57	0.57	0.57	0.57	0.58	0.61	0.36	0.37	0.37	0.37	0.37	0.39
**Genomic**												
G	0.65	0.54	0.49	0.44	0.40	0.38	0.52	0.41	0.38	0.35	0.27	0.29
GH	0.74	0.69	0.66	0.60	0.55	0.49	0.60	0.54	0.52	0.48	0.39	0.38
GS	0.67	0.67	0.70	0.72	0.75	0.79	0.53	0.53	0.56	0.58	0.59	0.63
GHS	0.74	0.74	0.75	0.75	0.76	0.79	0.60	0.59	0.61	0.60	0.60	0.63

Table S7: Average CRPS for EBV and PBV with strong genetic connectedness, using pedigree or genomic data, with varying proportion of spatial variance. The standard error for all values had order of data 10^{-3}

	EBV						PBV					
$\sigma_s^2/(\sigma_s^2+\sigma_h^2)$	0	0.2	0.4	0.6	0.8	1	0	0.2	0.4	0.6	0.8	1
**Pedigree**												
G	0.180	0.329	0.555	0.701	0.720	0.731	0.187	0.247	0.331	0.363	0.375	0.389
GH	0.171	0.234	0.288	0.336	0.383	0.499	0.179	0.189	0.217	0.239	0.267	0.324
GS	0.174	0.179	0.178	0.180	0.182	0.180	0.184	0.165	0.164	0.165	0.164	0.159
GHS	0.170	0.178	0.175	0.177	0.181	0.179	0.179	0.162	0.160	0.161	0.162	0.159
**Genomic**												
G	0.139	0.188	0.237	0.289	0.337	0.394	0.155	0.173	0.211	0.243	0.289	0.313
GH	0.122	0.134	0.149	0.176	0.205	0.264	0.143	0.132	0.142	0.160	0.188	0.221
GS	0.136	0.135	0.130	0.126	0.120	0.111	0.154	0.130	0.127	0.125	0.122	0.115
GHS	0.122	0.122	0.120	0.120	0.117	0.111	0.143	0.122	0.120	0.122	0.120	0.115

Changing the herd clustering

Table S8 and Table S9 respectively show average accuracy and CRPS between TBV and EBV/PBV for all levels of genetic connectedness, using both pedigree and genomic data, when the herd locations were simulated from a bi-variate normal distribution with mean equal to the village locations, and variance $1 \cdot 10^{-4}\mathbf{I}_{2\times2}$ (strong herd clustering). Table S10 and Table S11 respectively show average accuracy and CRPS between TBV and EBV/PBV for all levels of genetic connectedness, using both pedigree and genomic data, when the herd locations were simulated from a bi-variate normal distribution with mean equal to the village locations, and variance $9 \cdot 10^{-4}\mathbf{I}_{2\times2}$ (weak herd clustering).

Table S8: Average accuracy for the different levels of genetic connectedness for EBV and PBV, using pedigree or genomic data, and the herd locations simulated using variance $1 \cdot 10^{-4}\mathbf{I}_{2\times2}$ (strong herd clustering). The standard error for some values had order of magnitude 10^{-2}, and most had 10^{-3}

	Weak		Intermediate		Strong	
	EBV	PBV	EBV	PBV	EBV	PBV
**Pedigree**						
G	0.32	0.27	0.32	0.18	0.32	0.19
GH	0.35	0.29	0.41	0.22	0.41	0.25
GS	0.51	0.48	0.56	0.34	0.55	0.35
GHS	0.53	0.50	0.58	0.36	0.57	0.37
GHSC	0.56	0.54	0.59	0.37	0.58	0.38
**Genomic**						
G	0.32	0.30	0.40	0.29	0.42	0.32
GH	0.34	0.32	0.51	0.38	0.58	0.46
GS	0.57	0.55	0.70	0.54	0.72	0.57
GHS	0.61	0.58	0.73	0.58	0.75	0.60
GHSC	0.63	0.60	0.74	0.59	0.75	0.61

Table S9: Average CRPS for different genetic connectedness for EBV and PBV, using pedigree or genomic data, and the herd locations simulated using variance $1 \cdot 10^{-4}\mathbf{I}_{2\times2}$ (strong herd clustering). The standard error for all values had order of magnitude 10^{-3}

	Weak		Intermediate		Strong	
	EBV	PBV	EBV	PBV	EBV	PBV
**Pedigree**						
G	0.559	0.438	0.667	0.406	0.706	0.371
GH	0.419	0.374	0.343	0.281	0.335	0.252
GS	0.168	0.168	0.166	0.180	0.180	0.183
GHS	0.165	0.164	0.166	0.178	0.179	0.181
GHSC	0.160	0.159	0.163	0.176	0.176	0.179
**Genomic**						
G	0.395	0.402	0.325	0.302	0.299	0.264
GH	0.372	0.378	0.225	0.222	0.180	0.181
GS	0.152	0.156	0.130	0.151	0.126	0.146
GHS	0.147	0.151	0.124	0.146	0.120	0.142
GHSC	0.143	0.147	0.123	0.145	0.120	0.142

Table S10: Average accuracy for the different levels of genetic connectedness for EBV and PBV, using pedigree or genomic data, and the herd locations simulated using variance $9 \cdot 10^{-4}\mathbf{I}_{2\times 2}$ (weak herd clustering). The standard error for some values had order of magnitude 10^{-2}, and most had 10^{-3}

	Weak		Intermediate		Strong	
	EBV	PBV	EBV	PBV	EBV	PBV
**Pedigree**						
G	0.33	0.29	0.32	0.17	0.32	0.19
GH	0.37	0.31	0.41	0.22	0.42	0.25
GS	0.55	0.54	0.56	0.34	0.55	0.35
GHS	0.56	0.55	0.59	0.37	0.57	0.37
GHSC	0.58	0.57	0.59	0.37	0.58	0.37
**Genomic**						
G	0.34	0.31	0.40	0.27	0.44	0.33
GH	0.36	0.33	0.53	0.38	0.61	0.47
GS	0.61	0.60	0.70	0.54	0.72	0.57
GHS	0.65	0.63	0.74	0.57	0.75	0.59
GHSC	0.67	0.65	0.74	0.58	0.75	0.60

Table S11: Average CRPS for the different levels of genetic connectedness for EBV and PBV, using pedigree or genomic data, and the herd locations simulated using variance $9 \cdot 10^{-4}\mathbf{I}_{2\times 2}$ (weak herd clustering). The standard error for all values had order of magnitude 10^{-3}

	Weak		Intermediate		Strong	
	EBV	PBV	EBV	PBV	EBV	PBV
**Pedigree**						
G	0.500	0.410	0.615	0.392	0.688	0.370
GH	0.393	0.348	0.326	0.270	0.325	0.248
GS	0.164	0.163	0.165	0.179	0.180	0.184
GHS	0.160	0.158	0.163	0.176	0.178	0.181
GHSC	0.156	0.155	0.161	0.175	0.177	0.180
**Genomic**						
G	0.374	0.387	0.308	0.289	0.282	0.253
GH	0.349	0.362	0.209	0.211	0.169	0.174
GS	0.145	0.147	0.130	0.152	0.126	0.147
GHS	0.137	0.141	0.123	0.146	0.119	0.143
GHSC	0.135	0.138	0.122	0.146	0.119	0.143

Correlation between true spatial effect and EBV with changing herd clustering

Table S12 shows the average correlation between the EBV and the true spatial effect for all levels of genetic connectedness, using pedigree or genomic data, when the herd locations were simulated from a bi-variate normal distribution with mean equal to the village locations, and variance $1 \cdot 10^{-4}\mathbf{I}_{2\times2}$ (strong herd clustering).

Table S13 shows the average correlation between the EBV and the true spatial effect for all levels of genetic connectedness, using pedigree or genomic data, when the herd locations were simulated from a bi-variate normal distribution with mean equal to the village locations, and variance $3.5 \cdot 10^{-4}\mathbf{I}_{2\times2}$ (intermediate herd clustering). This is an extended table from the main paper where model GHSC is included.

Table S14 shows the average correlation between the EBV and the true spatial effect for all levels of genetic connectedness, using pedigree or genomic data, when the herd locations were simulated from a bi-variate normal distribution with mean equal to the village locations, and variance $9 \cdot 10^{-4}\mathbf{I}_{2\times2}$ (weak herd clustering).

Table S12: Average correlation between EBV and true spatial effect for all levels of genetic connectedness, using pedigree or genomic data, and the herd locations simulated using variance $1 \cdot 10^{-4}\mathbf{I}_{2\times2}$ (strong herd clustering). The standard error for all values had order of magnitude 10^{-3}

Connectedness	Weak	Intermediate	Strong
**Pedigree**			
G	0.87	0.76	0.70
GH	0.86	0.65	0.51
GS	0.23	0.06	0.03
GHS	0.27	0.07	0.03
GHSC	0.21	0.06	0.03
**Genomic**			
G	0.67	0.64	0.63
GH	0.71	0.62	0.60
GS	0.12	0.07	0.06
GHS	0.13	0.06	0.06
GHSC	0.10	0.05	0.05

Table S13: Average correlation between EBV and true spatial effect for all levels of genetic connectedness, using pedigree or genomic data, and the herd locations simulated using variance $3.5 \cdot 10^{-4}\mathbf{I}_{2\times2}$ (intermediate herd clustering). The standard error for all values had order of magnitude 10^{-3}

Connectedness	Weak	Intermediate	Strong
**Pedigree**			
G	0.68	0.64	0.64
GH	0.70	0.60	0.58
GS	0.11	0.06	0.06
GHS	0.12	0.06	0.06
GHSC	0.10	0.06	0.06
**Genomic**			
G	0.84	0.74	0.69
GH	0.83	0.63	0.50
GS	0.16	0.05	0.04
GHS	0.21	0.05	0.04
GHSC	0.18	0.05	0.03

Table S14: Average correlation between EBV and true spatial effect for all levels of genetic connectedness, using pedigree or genomic data, and the herd locations simulated using variance $9 \cdot 10^{-4}\mathbf{I}_{2\times 2}$ (weak herd clustering). The standard error for all values had order of magnitude 10^{-3}

Connectedness	Weak	Intermediate	Strong
**Pedigree**			
G	0.83	0.72	0.67
GH	0.81	0.59	0.47
GS	0.12	0.04	0.03
GHS	0.15	0.04	0.03
GHSC	0.14	0.04	0.02
**Genomic**			
G	0.69	0.65	0.63
GH	0.67	0.57	0.56
GS	0.09	0.05	0.05
GHS	0.09	0.05	0.05
GHSC	0.08	0.04	0.04

Real data

For the models G, GH, GS and GHS applied to the full real data, we present the DIC in Table S15.

Table S15: DIC for models G, GH, GS and GHS applied to the full real data

Model	DIC
G	67329
GH	70964
GS	70096
GHS	70929
